# Safety and efficacy of the VenaTech™ Retrievable inferior vena cava filter: a first-in-man single-center prospective study

**DOI:** 10.1186/s42155-022-00325-y

**Published:** 2022-10-04

**Authors:** Carole Déan, Young Il Kim, Olivier Sanchez, Nicolas Martelli, Marc Sapoval, Oliver Pellerin

**Affiliations:** 1 Université de Paris-Cité, PARCC, INSERM-970, Department of Interventional Radiology Assistance Publique - Hôpitaux de Paris - Hôpital Européen Georges-Pompidou, 20 Rue Leblanc, F-75015 Paris, France; 2 Université de Paris-Cité, IThEM INSERM UMR-S 1140, Department of Pneumology and Intensive Care Hôpital Européen Georges-Pompidou Assistance Publique - Hôpitaux de Paris, F-75015 Paris, France; 3 Université Paris-Saclay GRADES, Department of Pharmacy, Assistance Publique - Hôpitaux de Paris - Hôpital Européen Georges-Pompidou, F-75015 Paris, France

**Keywords:** Inferior vena cava filters, Pulmonary embolism, Deep vein thrombosis, Interventional imaging, Prospective study, First-in-man

## Abstract

**Background:**

Venous thromboembolism (VTE) is a frequent condition worldwide, associated with significant morbidity and mortality. Though its primary treatment is anticoagulation, the placement of an inferior vena cava (IVC) filter is recommended in patients with some comorbidities. The objectives of this study were to evaluate the clinical safety and efficacy of the Venatech® retrievable IVC filter. This open-label prospective single-center study was conducted on 40 consecutive patients requiring temporary or permanent IVC filtration. Patient characteristics, technical success rates of filter placement and removal, and the occurrence of complications were assessed. Follow-up imaging was performed using CT-scan before retrieval or at 6 months in the permanent indication population.

**Results:**

The filter was successfully implanted at the intended location in all the patients. Retrieval was attempted in 21 (52.5%) patients after a mean period of 50 days (range: 6–94 days), and the filter was successfully removed in 18 patients (85.7%). Reason for retrieval failure was filter with trapped thrombus (*n* = 2) and a > 15° tilt (*n* = 1). No complication was observed during the filter placement and retrieval. Follow-up imaging available in 30 patients (75%) demonstrated deep filter penetration (> 3 mm) in four patients (13.3%), severe filter tilt (> 15^o^) in five patients (16.7%), filter with trapped thrombus in three patients (10%), but no fracture or IVC thrombosis.

**Conclusion:**

This prospective study showed encouraging preliminary results of the safety and efficacy of the Venatech® retrievable IVC filter. The filter was easily delivered in the intended position and successfully removed in a high percentage of patients.

**Trial registration:**

ClinicalTrials.gov Identifier: NCT02674672

## Background

Venous thromboembolism (VTE) which includes both deep vein thrombosis (DVT) and pulmonary embolism (PE) is a frequent condition worldwide, associated with significant morbidity and mortality (Benjamin et al. 2019). Though its primary treatment is anticoagulation, the placement of an inferior vena cava (IVC) filter is recommended in patients: with acute DVT and / or PE with a contraindication to anticoagulation; with a < 3 months VTE and a planned surgery at risk of bleeding and VTE (eg. gynecologic surgery) (Kaufman et al. 2020; Couturier et al. 2016). The permanent placement of IVC filters was shown to be effective to prevent recurrence of PE but it is associated with an increased risk of DVT and post-thrombotic syndrome without long-term benefits on survival (PREPIC Study Group 2005). To reduce the risks related to the prolonged presence of the filter in the IVC, it is recommended to use temporary filters which can be retrieved when IVC filtration is no longer required (Caplin et al. 2011). Nonetheless, retrievable filters can be converted to a permanent use if filtration is still required (Jia et al. 2018). The Venatech® Retrievable IVC filter is a new filter which can be retrieved if required within the 12 weeks after implantation.

The purpose of the study was to evaluate the safety and efficacy of the Venatech® Retrievable IVC filter in a first-in-man single-center non-randomized prospective trial.

## Results

### Study population

**Fig. 1:**
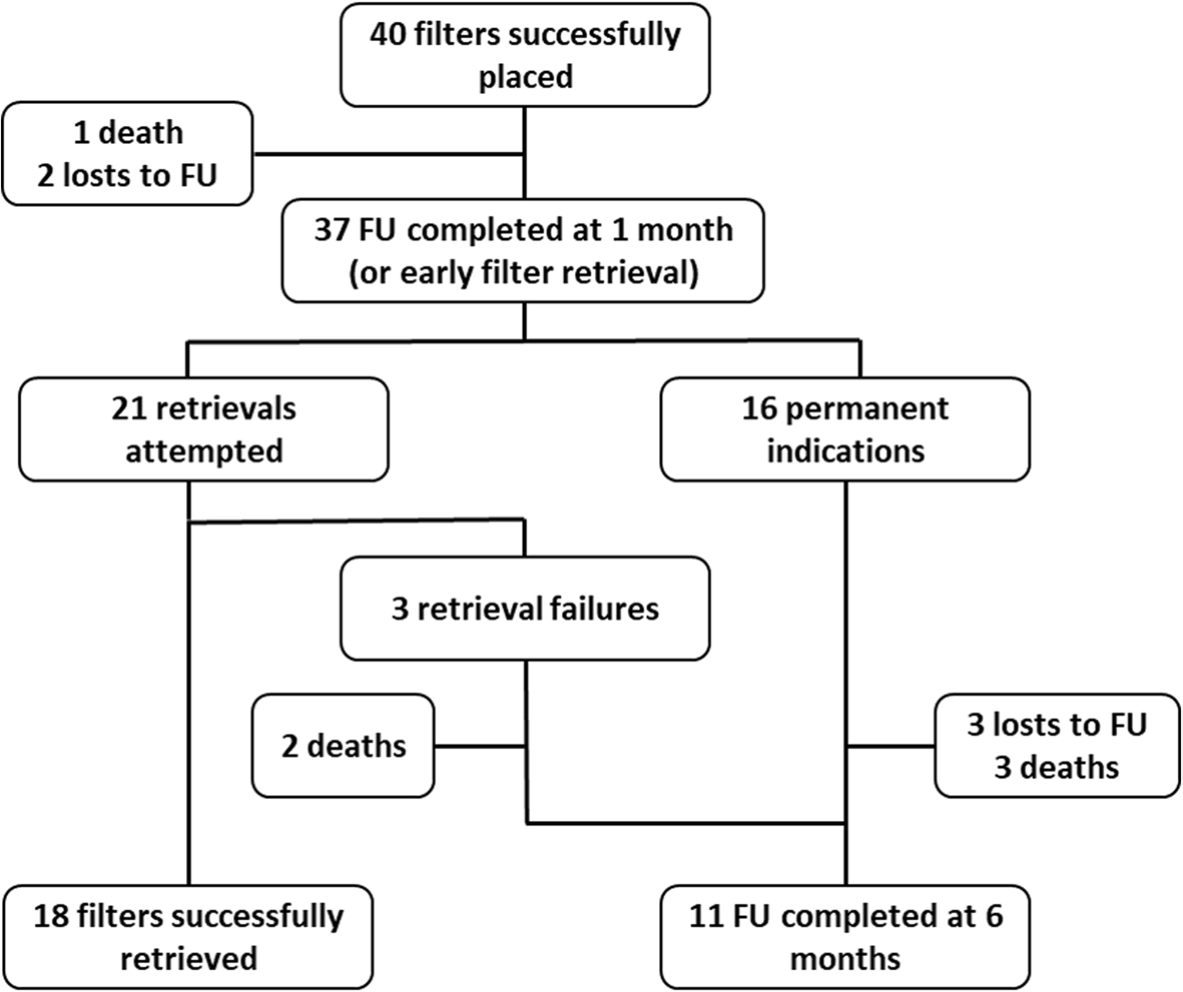
Study flowchart

A total of 40 consecutive patients (16 men/24 femal) with a mean age of 70 years [29–93 years] were included in the study.. All the patients were diagnosed for a DVT and 39 patients (97.5%) also had a concomitant PE (Table 1). Two patients (5%) were lost to follow-up after filter implantation and 3 patients (7.5%) were lost to follow-up after the one-month follow-up. Twenty-one patients (52.5%) had a filter retrieval attempted (temporary population), the remaining 16 patients were considered as “permanent population” since they still have a contra-indication to anticoagulation. Twenty-nine patients (72.5%) completed the study as required per-protocol, i.e. at filter successful retrieval or at the 6-month follow-up (permanent indication and in case of retrieval failure), with a mean follow-up of 105 ± 78 days (range: 6–267 days). Study flowchart is detailed in Fig. 1.

**Table 1:** Patient population

Data	Value
Age	70 ± 14 years (29–93)
Male/Female	16/24
Anticoagulation regimen before placement:	
None	3 (7.5)
Vitamin K antagonists	3 (7.5)
Direct oral anticoagulants	7 (17.5)
Low-Molecular-Weight Heparins	27 (67.5)
Indications for placement:	
Pulmonary thrombo-embolism with contraindication to anticoagulation^a^	30 (75)
Planned surgery with a high risk of both bleeding and pulmonary thrombo-embolism in patients with an < 90 days DVT / PE condition^b^	10 (25)
Failure of anticoagulant therapy in thrombo-embolic diseases	0
PE/DVT Risk factors:	
Major surgery scheduled within next 72 hours	10 (25)
Active cancer	16 (40)
Acute trauma	5 (12.5)
Prolonged immobilization	31 (77.5)
Hypertension	15 (37.5)
Current smoking	4 (10)
Obesity	9 (22.5)
Diabetes	3 (7.5)
Cardiopathy	8 (20)

Values are provided as Mean ± SD (range) or n(%)

^a^gastrointestinal, genito-urinary, spontaneous soft tissue, post-surgical bleeding, trauma, stroke

^b^gynecologic, orthopedic, gastrointestinal surgery

### Filter implantation

**Fig. 2:**
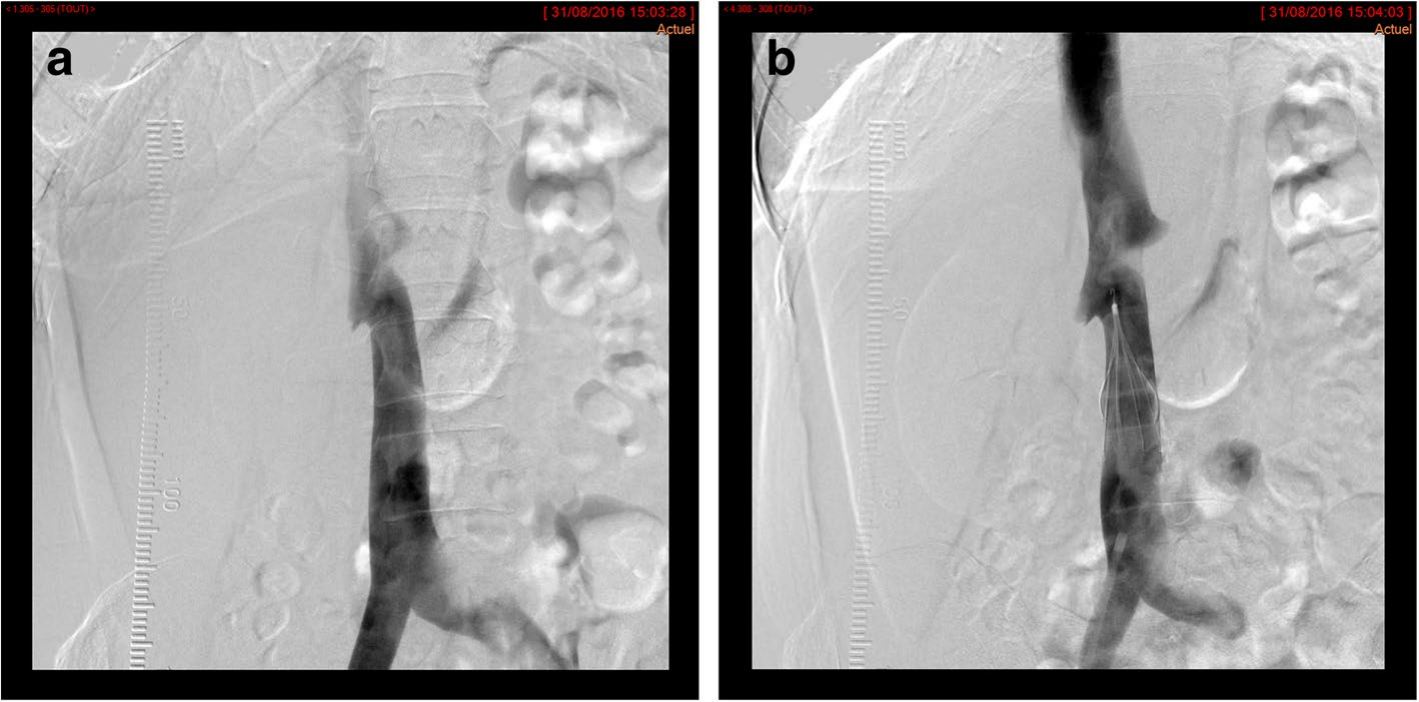
Anteroposterior angiography of the inferior vena cava before (**a**) and after filter implantation (**b**) for a 65 years old woman with current PE and DVT contra-indicated to anticoagulant therapy because of scheduled gynecological surgery (ovarian cancer). The angiography showed a fully patent inferior vena cava (**a**) and the filter was deployed bellow the lowest renal vein (**b**)

Procedural details are provided in Table 2. Filter insertion was performed by > 10 years’ experienced interventional radiologists via a right femoral (*n* = 29, 72.5%), a left femoral (*n* = 9, 22.5%) or a right internal jugular (*n* = 2, 5%) approach under local anesthesia and ultrasound guidance at puncture site. The access site was patent in all the cases. The mean infrarenal IVC diameter was 19.8 ± 3.8 mm (range: 15–27 mm), and the most frequent level chosen for deployment was L2 (Fig. 2). No duplicated IVC or anatomic abnormalities were described. Filter insertion was successfully performed at the intended site of the infrarenal IVC in all patients, therefore the primary technical success rate was 100%. The insertion was described by the interventional radiologists as easy in all the cases and no additional manipulation was required. A tilt was described at insertion in 14 cases (35%); it was > 15° in 3 cases (7.5%).

**Table 2:** Procedural data at implantation and retrieval

Data	Value
Level of deployment at insertion:	
T12	1 (2)
L1	10 (25)
L1/L2	2 (5)
L2	17 (42)
L2/L3	1 (2)
L3	6 (15)
L4	3 (7)
Anticoagulation regimen at retrieval:	
None	1 (5)
Direct oral anticoagulants	1 (5)
Low-Molecular-Weight Heparins	19 (90)
Reasons for moderate difficulty to retrieve:	
Anterior tilt	1 (5)
Tilt > 15°	2 (9)
IVC thrombus	1 (5)
Additional retrieval devices:	
Grasping device (ALN® kit)	3 (14)
10 Fr. manual aspiration catheter	1 (5)
Reasons for failure of retrieval:	
Posterior tilt	1 (5)
Filter thrombosis	2 (9)
Time to retrieval failure (days)	[68, 70, 72]

Values are provided as n(%) or [exact value]

### Filter retrieval

**Fig. 3:**
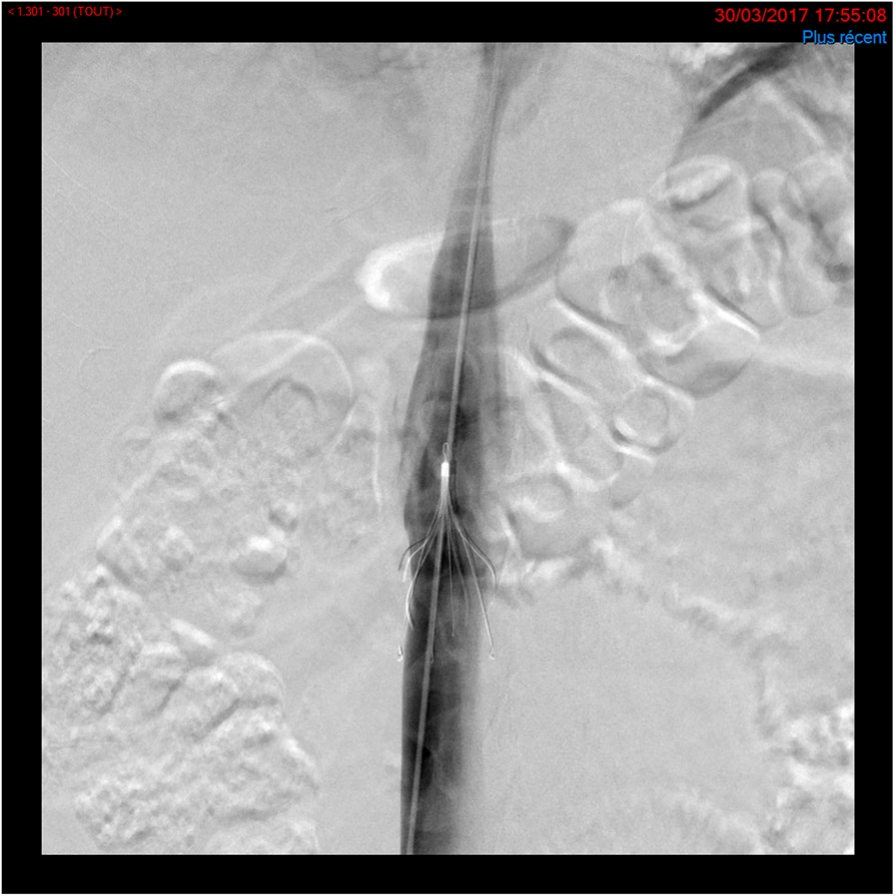
Anterioposterior view of snare manipulation to remove the filter. The patient was referred 90 days after implantation for filter removal. The cavogram showed a patent filter without tilt or filter struts vena cava penetration. A 20 mm snare was deployed over the filter hook. Since the filter was hooked, snare wire was pinned while the 13Fr catheter was advanced over the snare wire to collapse the filter. The filter was pulled into the 13fr catheter for extraction. The post extraction cavogram showed no abnormality.

Filter was planned to be retrieved as soon as the contra-indication to anticoagulation was waived. Retrieval procedures were performed by a > 10 years experienced interventional radiologist. Twenty-one patients (52.5%) underwent a filter retrieval attempt after a mean 50 ± 29 days (range: 6–94 days) post-implantation. Upon the 18 extracted filter, 14 (77.8%) were removed with a 20 mm snare (Fig. 3). A grasping device (ALN Implant Chirurgicaux, Bromes les Mimosas, France) was necessary to remove three filters, because all had > 15° tilt (respectively 23°; 32° and 33°). At last, one patient had first a manual aspiration of a trapped clot within the filter (10Fr. femoral access) and then filter removal with a20mm snare. In all cases, the final cavogram did not show evidence of vena cava injury.

Three removal procedure were withdrawn. Two of them because of a filter thrombosis without major tilting and the third one because of filter head embedding into the vena cava wall due to a tilt of 36°. Filter thrombosis was treated by oral anticoagulation.

### Permanent population follow-up

Mean follow-up was 140 ± 61 days (range: 33–267) in the permanent population (*n* = 19). No clinical PE recurrence was observed. Eleven patients (59.7%) completed to study and 10 patients (52.6%) had an IVC angioCT-scan imaging available at FU; complications are detailed in Table 3. One 30° tilt was associated with a 10 mm penetration in the IVC wall extending in the soft tissue. Other recorded 10 mm and 11 mm IVC penetrations also extended to the soft tissue but were not associated with a filter tilt. All struts IVC penetration were not clinically relevant. In this permanent population at the end of the study, 1 patient (5.3%) was under direct oral anticoagulants, another one patient (5.3%) was under Low-Molecular-Weight Heparins and the remaining 17 patients (89.4%) were not having any anticoagulant regimen. Six patients (15%) died during the study course with a mean delay from filter implantation of 90 ± 31 days (range: 6–138 days). All deaths were attributed to the normal evolution of the index illness. Four patients (66.7%) died from their active cancer, one patient (16.7%) with an acute major trauma died 6 days after filter implantation because of multiple organ failure and one patient died from the consequences of a liver and spleen trauma. No clinical events suggesting the recurrence of PE were observed during or after the filter placement in any patient.

**Table 3:** Filter feature assessed on angioCT-scan

Data	Temporary population	Permanent population
Number	21	10
Delay from implantation (days)	42 ± 25	146 ± 71
IVC occlusion	0	0
Filter thrombosis/embolization:	3 (14)	0
Filter fracture	0	0
Filter migration	0	0
Tilt:		
< 15°	2 (9)	2 (20)
> 15°	3 (14)	2 (20)
IVC wall penetration:		
< 3 mm	4 (19)	2 (20)
> 3 mm^a^	1 (5)	3 (30)

Values are provided as Mean ± SD (range) or n (%)

^a^IVC wall penetration were respectively 4 mm in the temporary population and, 4, 5 and 5 mm in the permanent population and were limited to fat surrounding the IVC

## Discussion

The use of retrievable IVC filters gained more spotlight than that of the permanent devices since their introduction into the clinical practice. The advantage of subsequent removal when no longer indicated against PE has been the main reason for the rising popularity of retrievable filters (Mismetti et al. 2015; Stavropoulos et al. 2016). **T**he Venatech® Retrievable is designed based on the Venatech® LP/LGM. The PREPIC study, a randomized controlled study, demonstrated the superiority of the Venatech® LP compared to anticoagulation to prevent PE and recurrence (PREPIC Study Group 2005). Thanks to its favorable design (self-centering without > 15° tilt, no device migration), the Venatech® LP efficacy profile is high. The VenaTech® Retrievable IVC filter consists in a flexible, symmetrical, self-expanding implantable filter and delivery accessories to accommodate delivery and implantation either via a femoral or jugular approach. According to the Instructions for Use, it can be retrieved within 12 weeks after implantation. It is CE-marked in Europe but not yet commercialized. This first-in-man single-center prospective study was designed to describe its safety and efficacy. The main results of the study are the good safety and efficacy of the device. Filter insertion was easy and successful in all the patients.

Filter retrieval was scheduled in 52.5% of the population, the other 47.5% of the patient still have indication of IVC filtration. The number of retrieval procedure is lower than the 64% and 62% reported respectively by Lin et al. and Stavropoulos et al. but could be due to the population including 40% of the patients having an active cancer (Stavropoulos et al. 2016; Lin et al. 2020). The retrieval success rate was high (86%) and the ease of retrieval was estimated by the interventional radiologist as easy in the majority of the cases. Theses good features are likely related to the conical design of the filter. Moreover, the large centering legs offer more contact to the vena cava wall reducing tilting.

Filter major complications stayed low. The rates of filter thrombosis (10%), tilt > 15° (16%) and penetration > 3 mm (13%) similar to other competitors and stay below recommended ranges (Jia et al. 2018; Comes et al. 2018; Gotra et al. 2018). In all the cases, the IVC wall perforation was asymptomatic and situated in the fat or the soft tissue. A major tilt prevented from filter retrieval in one case only. No filter migration or fracture were observed.

Concerning the anticoagulation regimen, the majority of the patient wasn’t under anticoagulation at follow-up the permanent population. In the temporary population, nearly all the patients were under an anticoagulant regimen at retrieval because the institution politics’ is to retrieve the filter as soon as the anticoagulant contra-indication is over. Therefore, anticoagulants were prescribed in the same time than the retrieval was scheduled. No clinical evidence of recurrence of PE or DVT at retrieval or as long as 6 months post-implantation were reported in the permanent population which is a sign of good efficacy compared to other devices (Bikdeli et al. 2017; Liu et al. 2021).

This study has several limitations. First, not all the patients had follow-up imaging and some patients were lost to follow-up. Second, the relatively short clinical follow-up time requires more studies to evaluate the long-term safety of the VenaTech® Retrievable IVC Filter.

To conclude, this first-in-man prospective study showed encouraging preliminary results of the safety and efficacy of the Venatech® retrievable IVC filter. The filter was easily delivered in the intended position and could be successfully removed in a high percentage of patients.

## Materials and methods

### Study design

The study was a prospective, open-label, stage 1 study performed in a single center (Sedrakyan et al. 2016). The sponsor was B.Braun Medical S.A.S (Chasseneuil, France). The study received appropriate Ethics Committee approval (CPP Ile-de-France VIII, Boulogne Billancourt, France; IDRCB: 2014-A01560–47) and informed consent was obtained from all patients prior to inclusion. B.Braun Medical designed the study under authors’ advice, gathered the data and decided to publish the paper. The authors analyzed the data, vouched for the data and analysis, and wrote the paper.

### Participants

All consecutive patients aged 18 years or older referred for an inferior vena cava filtration were screened. At least one of the following indications was required as inclusion criteria: VTE with contraindication to anticoagulation; failure of anticoagulant therapy in VTE; planned surgery with a high risk of both bleeding and VTE in patients with an < 90 days VTE condition. Main exclusion criteria were: vena cava with a diameter < 14 mm or > 28 mm; duplicated IVC; life-expectancy estimated < 6 months.

### Study device

**Fig. 4:**
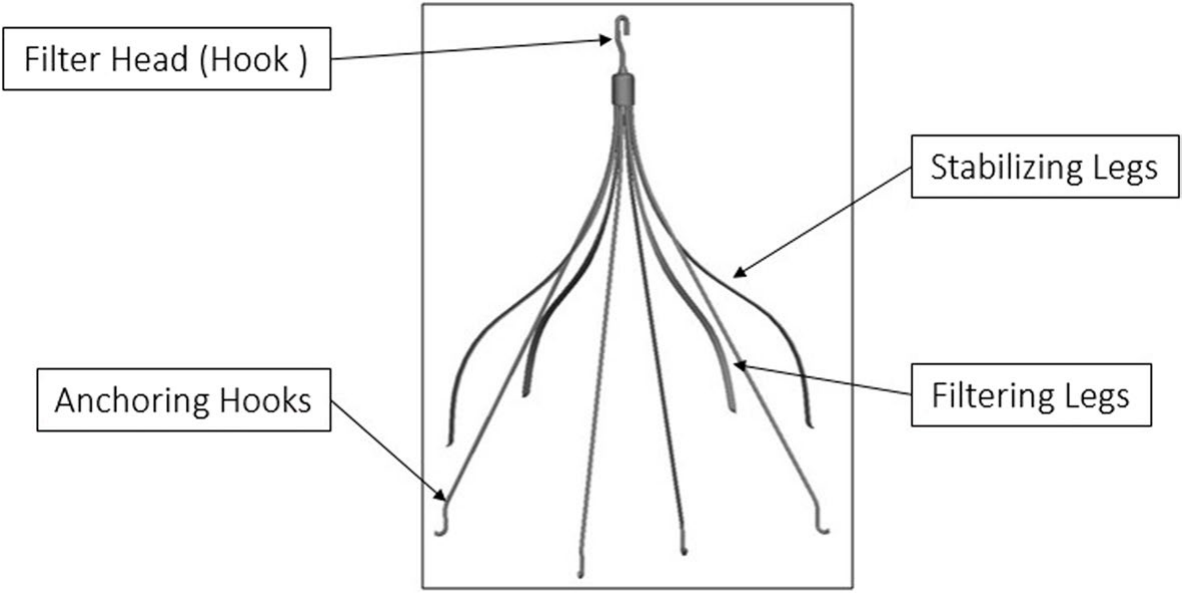
VenaTech® Retrievable Filter

The study device was the VenaTech® Retrievable Filter System (B.Braun Medical S.A.S.; Boulogne Billancourt, France). The filter has a specific design based on the previous B. Braun experience in caval filtration. The VenaTech® Retrievable filter is made of a chromium-cobalt alloy, MRI 3 Tesla compatible and CE marked FDA approved. It is cone shaped device made in 4 parts: stabilizing legs, filtering legs, a filter head and removal hook (Fig. 2). The stabilization legs and the filtering legs have anchoring hooks which securely position the filter in the center of the vena cava. The filtering legs are secured at the apex of the cone with a head that enables the filter to be retrieved. The VenaTech® Retrievable filter is pre-loaded in a cartridge and its delivery is performed though a 7Fr dedicated introducer sheath. Implantation can be performed either though femoral or jugular vein access. The IVC diameter must be between 14 mm and 28 mm in diameter (Fig. 3). The filter can be retrieved within the 12 weeks after implantation (temporary filtration) or used as a permanent filter. The removal procedure is performed by the manipulation of snare (eg.Amplatz GooseNeck® loop snare (ev3® Inc., Plymouth, MN)) inserted into a dedicated 13F sheath (Fig. 4). The sheath is advanced as close as the filter removal hook, then the snare is deployed over the hook. When the hook is captured by the snare loop a tension is exerted on the snare to align the main device axis, the snare and the 13 Fr sheath. To secure the filter capture, the snare caterer is slide over the wire loop. Afterward, the 13 Fr sheath is push over the snare to collapse filter legs. When the filter is completely retracted into the introducer sheath, the catheter snare is retracted.

### Implantation and retrievable technique

Before filter placement, an IVC venogram was obtained to confirm the patency of the vena cava, to identify any venous anomalies, to determine the location of the renal veins, and to evaluate antero-posterior and transverse diameter of the vena cava. The filter was implanted in an inferior vena cava with a diameter in-between 14 mm and 28 mm, below the lowest renal vein ostium.

Retrieval of the filter was scheduled after obtaining an IVC angioCT-scan to check the patency of the IVC and filter.

### Patients’ follow-up

All patients were followed at 1 month after implantation to decide the permanent or temporary indication for filtration unless the filter was already retrieved. For the “temporary filtration population”, retrieval was scheduled within the 12 weeks of implantation. Patients with a permanent indication of filtration were followed for 6 months after implantation. Follow-up imaging was obtained on an IVC angioCT-scan before retrieval or at the 6-month follow-up depending on the population. End of the study was the filter retrieval for the temporary filtration population or the 6-month follow-up for the permanent indication population and patients with a failure of retrieval.

### Study endpoints

The efficacy endpoints were the technical success for filter placement and retrieval which were respectively defined as the successful insertion and retrieval of the filter without any perprocedure complication. Adequate filter placement was defined as filter tip facing to the lower renal vein ostium, filter tilt lower than 15°and filter struts penetration lower than 3 mm outside the contrast column. Failure to filter placement was defined as the occurrence of any of the following: unsuccessful placement / withdrawal of the delivery system, failure to deploy and fix the filter at the intended position, whilst at the intended position, failure to deploy the filter, major filter movement occurring at deployment, filter cranial migration during deployment, incomplete filter opening in the IVC lumen, prolapse of filter component, filter fracture during placement, incomplete opening of the stabilizing legs or inadequate distribution of filtering legs. Technical success of retrieval was defined as the complete extraction of the filter from the vena cava with or without additional maneuvers. Any event occurring during the placement or retrieval procedure was recorded.

The safety of the filter following placement was based on the technical difficulties during filter deployment and retrieval and the occurrence of filter-related complications.

### Statistical analysis

No formal sample size calculation was done. A sample size of 40 subjects was considered as sufficient to evaluate the rate of complications related to the use of the VenaTech® Retrievable Filter System and to verify if unacceptable deviations occurred when compared to the recommended thresholds (Caplin et al. 2011). Statistical analysis was performed using R software (R Foundation for Statistical Computing, Vienna, Austria). Quantitative variables were expressed as mean ± standard deviation (SD) and range. Qualitative variables were expressed as raw numbers (n), proportion and percentages (%). The principal analysis was performed in the intention-to treat basis and included all evaluable patients.

## Data Availability

The data that support the findings of this study are available from BBraun but restrictions apply to the availability of these data, which were used under license for the current study, and so are not publicly available. Data are however available from the authors upon reasonable request and with permission of BBraun.

## Abbreviations

VTE: Venous thromboembolism
DVT: Deep vein thrombosis
PE: pulmonary embolism
IVC: Inferior vena cava
CT-scan: Computed tomography scanner
SD: Standard deviation
